# Effect of two dietary fibers on satiety and glycemic parameters: a randomized, double-blind, placebo-controlled, exploratory study

**DOI:** 10.1186/1475-2891-13-45

**Published:** 2014-05-17

**Authors:** David M Savastano, Rebecca J Hodge, Derek J Nunez, Ann Walker, Roxanne Kapikian

**Affiliations:** 1 Medical Affairs, Consumer Healthcare, GlaxoSmithKline, Parsippany, NJ, USA; 2 Discovery Medicine, GlaxoSmithKline, Five Moore Drive, MS N2-3208, Research Triangle Park, NC 27709, USA; 3 Statistics and Programming, GlaxoSmithKline, Research Triangle Park, NC, USA; 4 Biostatistics, Consumer Healthcare, GlaxoSmithKline, Parsippany, NJ, USA

**Keywords:** OFS, Pectin, Dietary fibers, Glucose, Satiety

## Abstract

**Background:**

Dietary carbohydrates may affect metabolic and physiologic parameters. The present study evaluated whether a combination of two dietary fibers, oligofructose (OFS) and pectin (P), altered satiety and glycemic parameters. The primary objective of this study was to determine whether dietary supplementation for 3 weeks with OFS + P would produce a greater reduction in energy intake of an *ad libitum* test meal compared to control.

**Methods:**

This was a single center, randomized, double-blind, placebo-controlled, parallel group study in overweight and obese, otherwise healthy, subjects (N = 96). There were two OFS + P treatment groups: high-dose (30 g/d), low-dose (15 g/d), and a control group (maltodextrin 15 g/d). Energy intake, appetite measures based on Satiety Labeled Intensity Magnitude (SLIM) scale, fasting and post-prandial glucose, and insulin levels and body weight were measured at baseline and at the end of 3 weeks. Adverse events and gastrointestinal tolerability of the treatments were also assessed.

**Results:**

An analysis of covariance (ANCOVA) performed on the primary endpoint change from baseline in energy intake, showed no statistically significant difference in energy intake among the three treatment groups (p = 0.5387). The LS mean changes (SE) in energy intake from baseline to week 3 were −58.3 (42.4) kilocalories (kcal) for the high dose group, −74.2 (43.6) kcal for the low dose group, and −9.0 (42.9) kcal for the control group. For the pairwise comparisons of OFS + P doses and control, confidence intervals were constructed around the difference in LS mean changes. All study products were generally well tolerated.

**Conclusion:**

There was a directional benefit in *ad libitum* energy intake for both OFS + P doses compared to control, with a greater reduction in kilocalories in the low dose comparison, but the reductions were not significant. Further studies are warranted.

**Clinical trial registration:**

GSK Clinical Study Register # W7781293

## Background

Satiety can be broadly defined as the feeling of fullness and/or the inhibition of hunger sensations after a meal. Appetite regulation has numerous determinants, including food composition, digestion, gastric emptying, and nutrient absorption, which together influence postprandial satiety responses. Various types of dietary carbohydrates differ considerably in the effects they exert on metabolic and physiologic parameters such as the postprandial glucose and hormone responses, gastric emptying, and intestinal transit time. The term ‘dietary fiber’ encompasses a variety of compounds that reach the colon undigested, including insoluble fibers such as wheat bran, soluble fibers from oats and fruits, resistant starches and oligosaccharides. The effect that a specific fiber has on satiety depends on its physical properties when eaten, how it is metabolized in the gut and its resultant physiological effects in the gut and elsewhere. In most cases, the physiologic benefits of a fiber can be further defined by considering solubility and viscosity. Viscosity is evaluated by how much the fiber thickens when it is added to fluid; it is also associated with water-holding properties. Several types of viscous fibers increase satiety by increasing stomach distension which can slow gastric emptying [1]. Another possible mechanism by which fibers increase satiety is through fermentation in the gut by microflora [2] and the subsequent effects of short-chain fatty acids (SCFA) produced. SCFA interact with G-coupled protein receptors such as GPR41 and GPR43 on enteroendocrine cells [3] and may be part of the mechanism for the effect of fiber on appetite as they increase production of satiety-related hormones from the colon [4-6].

Pectin (P) and oligofructose (OFS) are hypothesized to evoke satiety via SCFA production in the colon [7]. A small number of placebo-controlled trials demonstrated effects of OFS supplementation on satiety, satiety-related hormones, glycemic parameters, and weight loss in humans [8-12], although other clinical studies have demonstrated mixed or minimal effect [13]. Some clinical studies also suggest that pectin alters satiety, satiety-related hormones, glycemic parameters, and gastric emptying [14-19]. No clinical studies have explored the efficacy of a combination of OFS and pectin on these endpoints.

This study examined whether dietary supplementation with a combination of OFS and pectin (OFS + P) produces greater changes in satiety and glycemic parameters than a control oligosaccharide, maltodextrin (CON). Two dose levels (“high” and “low”) of OFS + P were tested. Measures of gastrointestinal tolerability and adverse events (AEs) were also evaluated.

## Methods

### Study subjects

Ninety-six healthy volunteers aged 18–60 years, with a BMI of 25.0 to < 35.0 kg/m^2^, consuming a usual diet of three main meals (5–7 days/week) at the time of enrollment, and who showed willingness to consume the required food, were recruited to participate in this study. The main exclusion criteria included pregnancy or breast-feeding, use of hormonal contraception, and history of gastrointestinal disease having an impact on food absorption or digestion, anorexia nervosa or bulimia nervosa, hypoglycemia, cardiovascular disease, elevated plasma glucose, unstable thyroid function, abnormal/irregular menstrual cycle, substance abuse, or being on medication having an effect on appetite. Subjects were also excluded if they were participating in any weight altering program, had a weight gain or loss of > 5 kg in the 3 months prior to enrollment, or had a history of intestinal discomfort when consuming relatively small (e.g., < 10 g) amounts of non-digestible carbohydrates and/or fibers.

The subjects were enrolled and study completed between October and December, 2011, at Biofortis-Provident Clinical Research, Addison, IL. Ethical approval for the study was obtained from Quorum Review Institutional Review Board (Reference no: QR# 26302/1). All participants gave written informed consent before entering the study. The study adhered to the principles of Declaration of Helsinki, ICH Good Clinical Practice, and other applicable regulations.

### Study design

**Figure 1:**
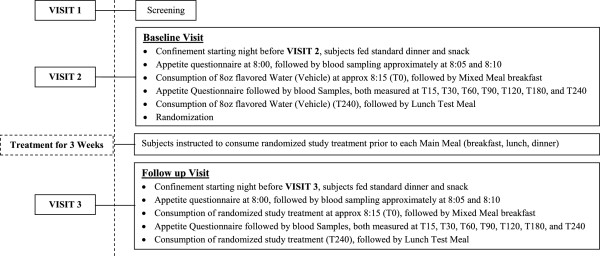
Study schematic.

This was a single-center, randomized, double-blind, placebo-controlled, parallel-group study in overweight and obese subjects, consisting of three separate clinic visits. Subject eligibility was determined at Visit 1 (screening visit). See Figure 1 for a study schematic.

Subjects who qualified for participation returned for Visit 2 (within 14 days of the screening visit). After reassessment of the inclusion/exclusion criteria, the eligible subjects underwent baseline evaluations for energy intake, subjective measures of appetite, as well as glycemic parameters. At this visit, subjects arrived at the site in the evening (no later than 17:00) prior to testing. They were provided with a standardized dinner (portion sizes based on calculated energy needs), to be consumed *ad libitum*, no later than 18:30, after which they completed an Eating Inventory. The Eating Inventory consists of 51 items and measures three dimensions of eating motivation: restraint, disinhibition, and hunger [20]. Subjects were confined to the site until the end of the test meal the next day, and were restricted from consuming any food after 21:30, but they could have water *ad libitum* until 07:00 the following morning. At approximately 07:30 (±10 min), fasting body weight was collected and an indwelling venous catheter was placed in the peripheral vein. Around 08:00 (±10 min), subjects completed a subjective appetite questionnaire, followed within 5 minutes by collection of a fasting blood sample to assess baseline concentrations of glucose and insulin. A second fasting blood sample was collected 4 min (±1 min) later to measure insulin levels for the calculation of the Homeostasis Model Assessment Insulin Resistance (HOMA-IR) score using the mean of the two fasting insulin values. At T0 (where “T” equals time and “0” equals zero min), five minutes after the second fasting blood sample, subjects consumed 8 oz (=240 ml) of room temperature water (vehicle) flavored with a commercially available water flavoring product (MiO®) (vehicle), followed by the mixed meal breakfast, consisting of a nutritionally balanced meal bar and a mixed fruit cup. At each time point, 15, 30, 60, 90, 120, 180 and 240 minutes, subjects completed the appetite questionnaire and a blood sample was collected. Subjects then consumed 8 oz of room temperature flavored water (vehicle) followed by the lunch test meal within 30 min. Lunch consisted of a single dish of cheese tortellini and pasta sauce to permit calculation of energy intake based on weight of food consumed.

At the end of Visit 2, subjects were randomized to one of three treatments (high-dose OFS + P, low-dose OFS + P, or maltodextrin control) to be consumed three times a day at meals. The randomization code was generated by the Biostatistics department of GSK Consumer Healthcare using a random number generator, and subjects were assigned to treatment by the investigative site in order of enrollment. The high-dose OFS + P contained 5.0 g OFS and 5.0 g pectin, and the low-dose OFS + P contained 2.5 g OFS and 2.5 g pectin. Maltodextrin (2.5 g + 2.5 g) was the placebo control in this study. The study treatments were provided as two individual sachets affixed together, and were added to approximately 8 oz of cold/room-temperature water and blended in a reusable ‘shaker’ bottle with a wire ball that was provided to the subjects. A total of 66 doses (2 sachets × 3 doses, 22 days of the assigned treatments) were provided to the subjects. For three weeks the subjects prepared the study treatment before each meal, consumed it within approximately 5 min, and documented the treatment compliance on a daily diary card that subjects returned on the last visit.

Three weeks later, at Visit 3, the eligibility criteria were reconfirmed, and Visit 2 procedures were repeated using the study treatments instead of vehicle. At this visit, the daily diary card and unused products were returned by the subjects. AE data were collected by subject diary or by self-report during the scheduled clinic visits of the study and gastrointestinal tolerability was evaluated at Visit 3 on the basis of a standardized questionnaire.

The commercially available water flavoring product was provided to subjects at the time study treatment was dispensed. Subjects were required to use the flavoring product (approximately ½ teaspoon) in the clinic (Visits 2 and 3) to help mask the active and control treatments and they were encouraged to use it during the 3 weeks of dosing to improve compliance with treatment.

Packaging and labeling of all study products was carried out according to current good manufacturing practices (cGMP) guidelines. The study products were packaged and labeled by Catalent Pharma Solutions, Philadelphia, PA., and prepared/managed by the GSK Clinical Supplies department. The clinical supplies were stored in a secure, temperature-controlled area with access limited to the investigator and authorized site personnel. Storage temperature was maintained between 59° and 86°F (15° and 30°C).

### Statistics

#### Sample size

As no clinical efficacy studies examining the combination of OFS + P on satiety have been reported, no formal sample size calculation was performed. The sample size chosen was comparable to those used in published placebo-controlled trials demonstrating effects of OFS supplementation on satiety and weight loss in humans [8-12]. A sufficient number of healthy subjects were screened by the study site to ensure approximately 96 subjects were randomized in order to yield at least 72 fully evaluable subjects, with approximately 24 subjects per treatment group.

#### Study populations

All randomized subjects who consumed the study treatment were considered evaluable for the safety population. The intention-to-treat (ITT) population included all randomized subjects who consumed the study treatment and had energy intake assessments at the test meal at baseline and week 3. The per-protocol (PP) population included all ITT subjects who completed the study without any major protocol deviations. Subjects were excluded from the PP population if they did not meet inclusion/exclusion criteria or took prohibited medication or product prior to or during the study treatment, or did not comply with product usage (took fewer than 54 doses of study product).

#### Efficacy variables

##### Primary efficacy parameter

The primary efficacy endpoint was the change from baseline in energy intake (kcal), defined as the difference between the test meal intake at 3 weeks and baseline, in the active groups compared to the control. The change from baseline to week 3 in energy intake was determined from the weight of the standardized test meals consumed by the subjects at lunch-time. At the test meal, the food was weighed prior to and following the meal to determine the total food intake, first at the baseline visit and then at the week 3 visit. Total food intake, recorded in grams (g), was converted to kilocalories (kcal) to derive the energy intake (conversion factor of 2.019 kcal/g, based on a 2120 kcal meal). The difference in energy intake between the baseline and week 3 visits was then calculated for each subject.

##### Secondary efficacy parameters

The secondary efficacy parameters in this study included: subjective appetite ratings, fasting and post-prandial glucose, and insulin values expressed as area-under-the-concentration curve 0-4 h (AUC_0–4_ h), and body weight.

The subjective appetite ratings were evaluated on the basis of Satiety Labeled Intensity Magnitude (SLIM) scale questionnaire [21]. The SLIM scale was rated on a 100 mm scale, from “greatest imaginable fullness” to “greatest imaginable hunger”. The SLIM scale rated the degree of hunger/fullness a subject felt at the point in time that the measurement was taken, namely at times 0, 15, 30, 60, 90 120, 180, and 240 minutes. The AUC for the SLIM scale ratings was calculated using the linear trapezoidal method applied to the observed ratings in order to obtain a cumulative measurement of the treatment effect on appetite characteristics across time.

For glucose and insulin, AUC_0–4_ h was calculated for the baseline (Visit 2) and week 3 (Visit 3) assessments. Actual time, in hours, relative to the start of the flavored water intake (baseline) or study product intake (week 3) was used to calculate the AUCs. The AUC was calculated using the linear trapezoid method for the mixed-breakfast meal challenge glucose and insulin values.

Body weight was measured at baseline and at the week 3 visit, and the change from baseline to week 3 was calculated.

##### Statistical methods for primary and secondary parameters

Descriptive statistics were computed for the primary and secondary outcome variables. For the primary analysis, linear modeling using analysis of covariance (ANCOVA) explored the change from baseline in energy intake, with treatment group as a factor, and baseline energy intake as a covariate in the model. For the pair-wise comparisons in least square (LS) means of the energy intake variable between the active treatments and the control, 95% confidence intervals were calculated for these comparisons. If the overall treatment effect was significant for the primary efficacy variable, then a hierarchical testing procedure was planned to adjust for multiplicity, and corresponding p-values were calculated for the pair-wise comparisons.

The pre-treatment to post-treatment changes were summarized for the secondary variables. In addition, HOMA-IR [22] and the Matsuda Index [23] were calculated to assess insulin resistance and insulin sensitivity, respectively. ANCOVA was applied to the change from baseline variables of SLIM scale (AUC), glucose (AUC), insulin (AUC), and body weight, with treatment group as a factor, and baseline value of the specific parameter included as a covariate in the model. The pair-wise difference in the LS means between each active product and control product was calculated, and 95% confidence intervals (CI) were constructed for these differences.

## Results

### Study subjects

**Figure 2:**
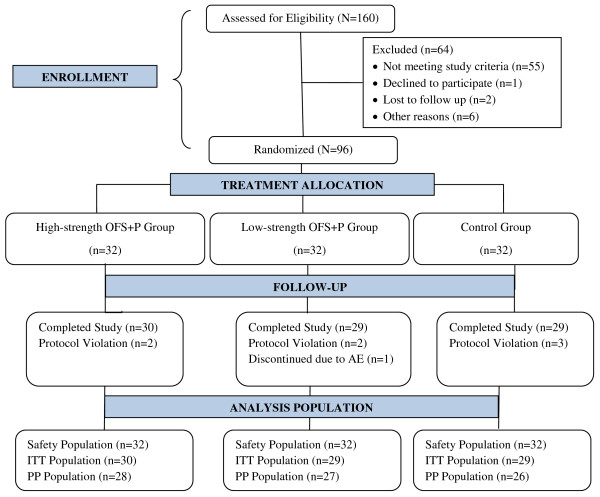
Subject disposition.

A total of 160 subjects were screened, of which 96 subjects were randomized to the study treatments. Subject disposition is provided in Figure 2. The ITT population included 88 (91.7%) subjects (30 in high-dose OFS + P group, 29 in low-dose OFS + P group, and 29 in control group) who had at least one post-randomization efficacy measurement. The PP analysis included 81 (84.4%) subjects (28 in high-dose OFS + P group, 27 in low-dose OFS + P group, and 26 in control group) who did not have any major protocol violations.

Overall, 51 (58.0%) females and 37 (42.0%) males constituted the ITT population of the study. The demographic and baseline characteristics of these subjects are provided in Table 1, and are comparable between the groups. The mean (SD) age of the enrolled subjects was 41.6 (12.4) years. The high-dose OFS + P group had a lower mean age and more females, compared to the other two groups. The majority of the enrolled population was Caucasian (70.5%). The mean (SD) body weight of the subjects was 83.1 (12.8) kg (Table 1).

**Table 1:** Demographic and baseline characteristics

**Parameter**	**High dose OFS + P (N = 30)**	**Low dose OFS + P (N = 29)**	**Control (N = 29)**	**Overall (N = 88)**
Age (years): Mean (SD)	39.2 (12.7)	43.4 (11.8)	42.3 (12.8)	41.6 (12.4)
Gender: n (%)				
Male	9 (30.0)	13 (44.8)	15 (51.7)	37 (42.0)
Female	21 (70.0)	16 (55.2)	14 (48.3)	51 (58.0)
Race: n (%)				
Asian	1 (3.3)	0	0	1 (1.1)
Black	6 (20.0)	7 (24.1)	11 (37.9)	24 (27.3)
Caucasian	23 (76.7)	22 (75.9)	17 (58.6)	62 (70.5)
Other	0	0	1 ( 3.4)	1 (1.1)
Body Weight (kg): Mean (SD)	81.4 (12.2)	82.1 (12.7)	85.3 (13.8)	83.1 (12.8)
BMI (kg/m^2^): Mean (SD)	29.3 (2.8)	28.3 (2.6)	29.4 (2.7)	29.0 (2.7)
Dietary fiber intake (g): Mean (SD)	17.1 (4.6)	16.9 (4.4)	16.3 (3.1)	16.8 (4.0)
Cognitive restraint of eating: Mean (SD)	8.9 (4.7)	8.2 (4.8)	8.7 (4.4)	8.0 (4.6)
Disinhibition: Mean (SD)	6.9 (3.8)	8.0 (4.3)	6.4 (3.5)	7.1 (3.9)
Hunger: Mean (SD)	6.6 (3.3)	7.4 (3.8)	5.9 (3.4)	6.6 (3.5)

An Eating Inventory, taken at baseline, was summarized by gender and overall for each treatment group. For the three measurements, cognitive restraint of eating, disinhibition, and hunger, there were slightly higher scores for females than males. The overall mean scores at baseline (minimum, maximum scores) across all the treatment groups were 8.6 [1,21] for the cognitive restraint of eating, 7.1 (0, 16) for disinhibition, and 6.6 (0, 14) for hunger.

Subjects were considered compliant if they took at least 54 of 66 doses of the study product. Compliance (%) was achieved by 29 (96.7%) subjects in the high-dose OFS + P group, 27 (93.1%) subjects in the low-dose OFS + P group, and 26 (89.7%) subjects in the control group, in the ITT population.

### Efficacy results

#### Energy intake at the test meal

The adjusted mean changes of energy intake at the test meal are summarized in Table 2. Overall, there was not a statistically significant difference among the treatment groups for energy intake (p = 0.5387). The LS mean changes (SE) from baseline to week 3 were −58.3 (42.4) kcal for the high-dose group, −74.2 (43.6) kcal for the low-dose group, and −9.0 (42.9) kcal for the control group. In the ITT population, the adjusted difference (95% CI) between the high-dose group and control was -49.3 (−168.8, 70.2) as compared to the difference between the low-dose group and control of −65.2 (−187.5, 57.0). Similarly, the difference among the treatment groups for energy intake for the PP population was not significant (p = 0.8265).

**Table 2:** Mean change from baseline to week 3: energy intake (Kcal) at the test meal

**Parameter**	**High dose OFS + P (N = 30)**	**Low dose OFS + P (N = 29)**	**Control (N = 29)**
Energy intake at baseline: Mean (SD)	694.4 (301.4)	896.3 (449.7)	732.9 (372.4)
Energy intake at week 3: Mean (SD)	648.3 (337.2)	803.3 (455.6)	730.3 (381.8)
Mean change^1^ (SD)	- 46.1 (236.7)	- 93.0 (260.8)	- 2.7 (208.7)
LS Mean change^2^	- 58.3 (42.4)	- 74.2 (43.6)	- 9.0 (42.9)
95% CI of LS mean change	- 142.6, 26.1	- 160.8, 12.4	- 94.2, 76.3
Difference from control^3^ (SE)	- 49.3 (60.1)	- 65.2 (61.5)	
95% CI of difference from control	- 168.8, 70.2	- 187.5, 57.0	

^1^Mean change was calculated as (Week 3 Mean–Baseline Mean).

^2^LS Mean Change adjusted for baseline energy intake effect, using analysis of covariance model.

^3^The difference from control is the difference in the LS Mean Changes of the active treatment minus the control. Overall treatment effect p-value = 0.5387.

#### Subjective ratings of appetite (SLIM scale)

**Figure 3:**
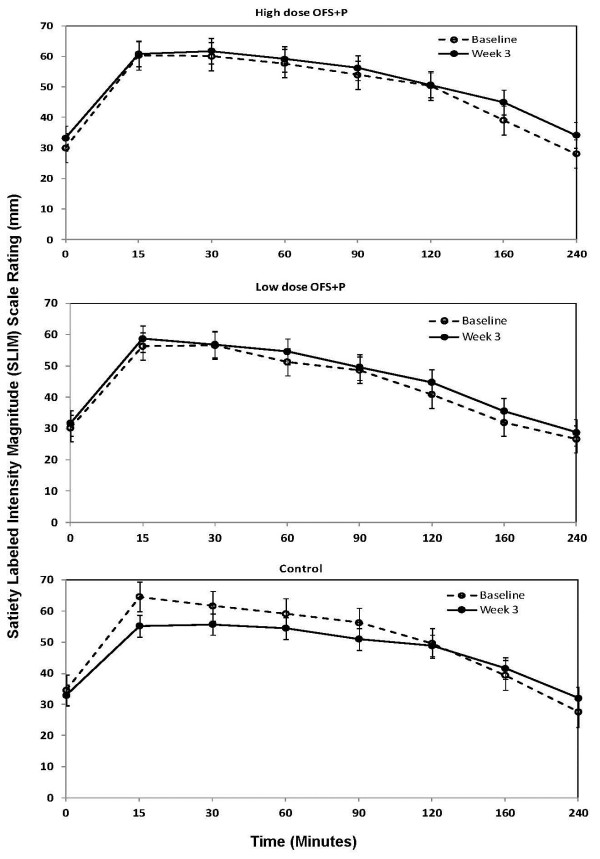
Mean (SEM) observed SLIM rating scale–time profile.

Appetite ratings increased in fullness from time 0 (fasting) to either 15 or 30 min post-meal, and then began to decrease until the final measurement at 4 hours, at both baseline and week 3. Mean scores of 50 indicate the subjects are neither hungry nor full, while values above 50 indicate degrees of fullness (highest degree is 100 = greatest imaginable fullness), and below 50 indicate degrees of hunger (lowest degree is 0 = greatest imaginable hunger). The mean scores crossed over the midpoint from fullness to hunger between 2.0 and 3.0 hours for the high-dose OFS + P group, between 1.0 and 1.5 for the low-dose OFS + P group, and between 1.5 and 2.0 for the control group, at both baseline and week 3. The observed appetite ratings are presented in Figure 3 for high-dose OFS + P, low-dose OFS + P and control.

Table 3 presents the AUC_0–4 hr_ results for the subjective ratings of appetite. Overall, there was not a statistically significant difference among the treatment groups (p = 0.1279). The LS mean change from baseline to week 3 was highest for the high-dose OFS + P group at 13.8, followed by the low-dose OFS + P group at 6.9, and then the control group at −3.7. The difference in LS mean change (95% CI) was 17.5 (0.5, 34.5) between the high-dose OFS + P group and the control and 10.6 (−7.0, 28.3) between the low-dose OFS + P group and the control.

**Table 3:** **Subjective ratings of appetite AUC**_**0−4**_ **hours**

**Parameter**	**High dose OFS + P (N = 30)**	**Low dose OFS + P (N = 29)**	**Control (N = 29)**
Baseline SLIM rating: Mean (SD)	188.1 (33.9)	164.9 (53.6)	191.9 (44.0)
SLIM rating at week 3: Mean (SD)	200.4 (39.2)	175.6 (50.1)	185.9 (52.2)
Mean change^1^ (SD)	12.4 (37.7)	10.8 (33.2)	- 6.0 (31.1)
LS Mean change^2^	13.8 (6.0)	6.9 (6.2)	- 3.7 (6.2)
95% CI of LS mean change	1.8, 25.8	- 5.5, 19.4	- 15.9, 8.6
Difference from control^3^ (SE)	17.5 (8.6)	10.6 (8.9)	
95% CI of difference from control	0.5, 34.5	−7.0, 28.3	

^1^Mean Change was calculated as (Week 3 Mean–Baseline Mean).

^2^LS Mean Change adjusted for baseline Slim Scale AUC_0-4hr_ effect, using analysis of covariance model.

^3^The difference from control is the difference in the LS Mean Changes of the active treatment minus the control. Overall treatment effect p-value = 0.1279.

#### Concentrations of blood glucose and insulin

**Figure 4:**
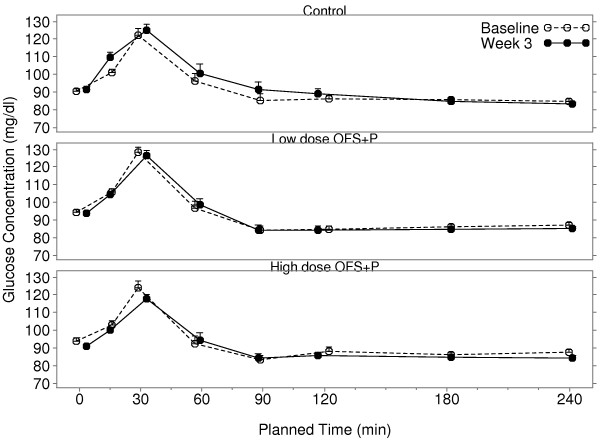
**Mean glucose concentrations fasting and over 4 h post-prandial at baseline and after 3 weeks of treatment.** Error bars represent the mean + SEM. OFS, oligofructose; P, apple pectin.

There was minimal change from baseline in fasting glucose in all three treatment groups (Table 4). However, after adjusting for baseline, the mean change from baseline (SE) in glucose AUC showed an increase of 8.5 (5.04) in the control group and reductions of 7.7 (4.95) and 3.0 (5.04) mg*hr/dL in the high and low dose OFS + P groups, respectively. The difference in the LS mean change from baseline (95% CI) was −16.2 (−30.2, −2.1) between the high-dose OFS + P group and control group and −11.5 (−25.6, 2.7) between the low-dose OFS + P group and control group. Figure 4 presents the mean glucose concentration-time profile curves of the three treatment groups.

**Table 4:** Fasting glucose, fasting insulin, HOMA IR and matsuda index

**Parameter**	**High dose OFS + P (N = 30)**	**Low dose OFS + P (N = 29)**	**Control (N = 29)**
**Fasting glucose (mg/dl)**			
Baseline mean (SD)	93.8 (10.41)	94.2 (8.55)	90.3 (10.00)
Week 3 mean (SD)	90.9 (7.95)	94.0 (7.71)	91.5 (9.58)
Week 3 LS mean change adjusted for baseline	−2.34	0.57	−0.06
LS Mean difference from control (95% CI)	−2.28 (−6.03, 1.46)	0.63 (−3.15, 4.42)	
**Fasting insulin (pmol/L)**			
Baseline geometric mean (SD log-scale)	37.5 (0.59)	40.6 (0.56)	40.4 (0.49)
Week 3 geometric mean (SD log-scale)	34.0 (0.52)	37.8 (0.54)	42.8 (0.61)
Week 3 geometric LS mean adjusted for baseline (SE log-scale)	35.4 (0.07)	37.0 (0.07)	42.0 (0.07)
Geometric LS mean ratio relative to control (95% CI)	0.84 (0.70, 1.01)	0.88 (0.73, 0.1.06)	
**Matsuda index**			
Baseline geometric mean (SD log-scale)	11.5 (0.56)	9.9 (0.55)	10.4 (0.52)
Week 3 geometric mean (SD log-scale)	11.7 (0.49)	10.2 (0.53)	8.8 (0.60)
Week 3 geometric LS mean adjusted for baseline (SE log-scale)	10.9 (0.06)	10.8 (0.06)	9.0 (0.06)
Geometric LS mean ratio relative to control (95% CI)	1.22 (1.04, 1.43)	1.21 (1.03, 1.42)	
	**(N = 25)**	**(N = 25)**	**(N = 26)**
**HOMA-IR**			
Baseline geometric mean (SD log-scale)	0.79 (0.506)	0.87 (0.422)	0.79 (0.447)
Week 3 geometric mean (SD log-scale)	0.71 (0.404)	0.78 (0.452)	0.91 (0.507)
Week 3 geometric LS mean adjusted for baseline (SE log-scale)	0.75 (0.067)	0.78 (0.067)	0.92 (0.066)
Geometric LS mean ratio relative to control (95% CI)	0.82 (0.68, 0.98)	0.85 (0.70, 1.02)	

For fasting insulin, HOMA-IR and Matsuda Index log-transformation was required to improve distributional properties. The dependent variable in the ANCOVA was the log of the week 3 result. The LS means were adjusted for log of baseline, using an analysis of covariance model.

**Figure 5:**
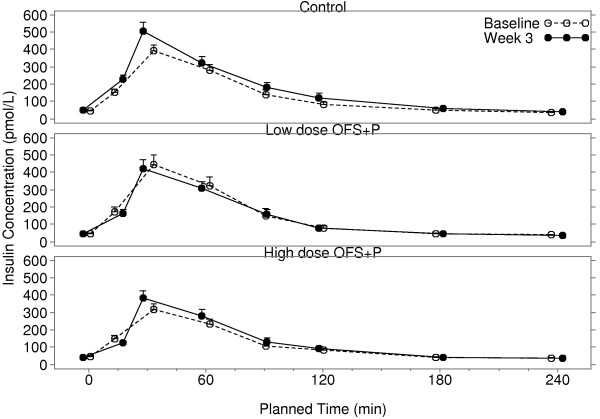
**Mean insulin concentrations fasting and over 4 h post-prandial at baseline and after 3 weeks of treatment.** Error bars represent the mean + SEM. OFS, oligofructose; P, apple pectin.

The week 3 adjusted geometric mean fasting insulin value was higher in the control group than both the high and low-dose OFS + P groups (Table 4). Relative to the control group, mean fasting insulin was reduced 16% in the high-dose OFS + P group (ratio: 0.84, 95% CI: 0.70, 1.01) and reduced 12% in the low dose OFS + P group (ratio: 0.88, 95% CI: 0.73, 1.06). Similarly, the week 3 adjusted geometric mean insulin AUC was higher in the control group, than in either the high or low dose OFS + P groups. Relative to the control group, mean insulin AUC was reduced by 10% in the high-dose OFS + P group (ratio: 0.90, 95% CI: 0.79, 1.04) and was reduced by 17% in the low-dose OFS + P group (ratio: 0.83, 95% CI: 0.73, 0.96). Figure 5 presents the mean insulin concentration-time profile curves of the three treatment groups.

A reduction in HOMA-IR may indicate a reduction in insulin resistance and an increase in the Matsuda Index may imply an increase in insulin sensitivity. Table 4 presents a summary for these parameters. The week 3 adjusted geometric mean HOMA-IR was higher in the control group (0.92), than in either the high dose (0.75) or low dose (0.78) OFS + P groups. Relative to the control group, mean HOMA-IR was reduced by 18% in the high dose OFS + P group (ratio: 0.82, 95% CI: 0.68, 0.98) and by 15% in the low dose OFS + P group (ratio: 0.85, 95% CI: 0.70, 1.02).

The week 3 adjusted geometric mean Matsuda Index was lower in the control group (9.0) than in either the high dose (10.9) or low dose (10.8) OFS + P groups. Relative to the control group, mean Matsuda Index was increased by 22% and 21% in the high and low dose groups, respectively (Table 4).

#### Body weight

Overall there was no statistically significant difference in body weight (measured in kg) among the three treatment groups (p = 0.1047). The difference in the LS mean changes from baseline (95% CI) was −0.6 (-1.1, −0.04) between the high-dose OFS + P group and control group and -0.3 (-0.8, 0.3) between the low-dose OFS + P and control group.

#### Adverse events

At least one AE was reported by 14 (43.8%) high dose OFS + P subjects, 9 (28.1%) low dose OFS + P subjects, and 8 (25.0%) control subjects. The most commonly reported AEs were flatulence, constipation, diarrhea, upper respiratory tract infection, and headache. The AEs were mild-moderate in intensity; also no serious AEs were reported by the subjects.

#### Gastrointestinal tolerability

A higher percentage of subjects experienced gas/bloating during the last 7 days of treatment in the high and low dose OFS + P groups (63.3% and 69.0%, respectively), as compared to the control group (34.5%). Similar results were reported for flatulence in the last 7 days of treatment, with 70% of the high dose subjects, 58.6% of the low dose subjects, and 44.8% of the control subjects experiencing this event.

A higher percentage of subjects reported diarrhea and gastrointestinal cramping in the last 7 days of treatment as occurring “somewhat more” and “much more than usual” in the high dose group, 33.3% and 26.7%, respectively, as compared to 10.3% and 13.8% of subjects in the low dose group.

Constipation in the last 7 days of treatment was experienced by more control subjects, 27.6%, as compared to 23.3% high dose subjects and 17.2% low dose subjects. Nausea in the last 7 days of treatment was reported by 1 or 2 subjects in each treatment group.

## Discussion

Given that obesity and its associated co-morbidities are at epidemic levels, it is relevant to examine whether combinations of dietary fiber or fiber supplements are effective therapies that promote satiety and reduce energy intake. The scientific literature documents several favorable effects of dietary fiber on calorie intake and glucose homeostasis. Previous clinical research suggests that OFS and pectin each produce effects on satiety, glycemic parameters, and weight loss in humans [8-19]. To our knowledge, this exploratory study is the first to examine whether dietary supplementation with a combination of OFS and pectin produces greater changes in satiety, glycemic parameters, and body weight compared to control. In general, there were no robust effects of the combination of OFS and pectin on energy intake of an *ad libitum* test meal, body weight, or subjective appetite ratings or glycemic parameters following a mixed meal challenge.

The baseline energy intakes differed among the three groups. The energy intake in the low dose OFS + P group was about 200 kcal higher as compared with the high dose group and about 160 kcal higher as compared with control. The change from baseline in energy intake showed a reduction over the three week period of 6.6% and 10.4% in the high and low dose OFS + P groups, as compared to 0.4% in the control group. Consequently, when adjusted for effects of baseline intake, both the high and low dose OFS + P groups demonstrated potentially meaningful reductions in energy intake, but the results were not statistically significant. Likewise, both the high and low dose OFS + P groups demonstrated increased overall ratings of fullness when adjusted for baseline effects, but the differences among the treatment groups were not significant. Additionally, there was large variability in energy intake and appetite ratings.There were no meaningful differences in changes from baseline in fasting glucose or insulin levels observed in any of the treatments. There were small placebo-corrected reductions in glucose AUC across breakfast for low and high dose OFS + P. However, as shown in Figures 4 and 5, the 5 g maltodextrin may have augmented the glucose and insulin excursions in the control group following the breakfast meal, in which 40 g of carbohydrate was consumed. There was little change in glucose and insulin AUC (0-4 h) with low dose OFS + P following the breakfast meal challenge, whereas there was a suggestion of a reduction of glucose AUC (0-4 h) with high dose OFS + P across the breakfast meal when compared to the low dose group. It is interesting to note that in the subset of subjects in the high dose group who actually had glucose reductions, insulin was also reduced in the majority of cases, suggesting improved glucose disposal with lower insulin levels (data not shown). This is consistent with the changes in HOMA-IR and Matsuda Index in the two OFS + P treated groups that indicate an improvement in insulin sensitivity relative to control. However, much of the difference is due to alteration of the glucose/insulin dynamics in the maltodextrin control group.

A small difference in the change from baseline body weight was observed between the high dose OFS + P and control groups at week 3. However this was primarily due to weight gain in the control group. The change from baseline in weight showed small increases over the three week period of 0.1% and 0.5% in the high and low dose OFS + P groups, as compared to 0.7% in the control group. Given that the high dose OFS + P and control treatments were matched for (estimated) metabolizable energy, this observation suggests that high dose OFS + P subjects may have compensated for the additional energy intake in the study treatment by eating less across the study period, whereas control subjects did not. Further study would be necessary to determine whether regular consumption of OFS + P supports body weight maintenance, or promotes weight loss when combined with a hypocaloric diet.

Overall, the results of this study do not provide strong support for the regular consumption of OFS + P for the short-term management of satiety. One possible explanation may be that the doses selected for either OFS or pectin were not optimal. For example, the doses selected in our study may not have been high enough since both 16 g of OFS [10] as well as 15 g of pectin [16] have each been shown to effectively increase the acute sensation of satiety in adult obese patients. Conversely, 5 g of pectin, as was used in our high-dose OFS + P group, produced satiety in healthy-weight men and women when assessed acutely [15].

The safety and tolerability results in the present study were consistent with what is expected from consumption of dietary fibers. The most commonly observed AEs were gastrointestinal in nature, none of which were serious.

## Conclusion

In conclusion, dietary supplementation of OFS + P tested at two doses did not produce statistically significantly greater reductions from baseline in energy intake of a test meal compared to the control. Although this pilot study did not demonstrate significant changes, we observed a directional benefit in *ad libitum* energy intake for high dose OFS + P and low dose OFS + P compared to control, with a greater reduction in kilocalories in the low dose comparison. Similarly, there may be improvement in insulin sensitivity. Further research on combinations of dietary fiber and other potential dietary therapies is warranted.

## Abbreviations

AE: Adverse Events; ANCOVA: Analysis of covariance; cGMP: Current good manufacturing practices; CON: Maltodextrin control; HOMA-IR: Homeostasis Model Assessment Insulin Resistance; ITT: Intent-to-Treat; LS: Least-Squares; OFS: Oligofructose; P: Apple pectin; PP: Per-protocol; SCFA: Short-chain fatty acids; SLIM Scale: Satiety Labeled Intensity Magnitude.

## Competing interests

The authors declare that they have no competing interests.

## Authors’ contributions

All authors contributed to conception and design of the study; A.W and R.K. analyzed the data; all authors contributed to the interpretation of the data, drafting of the article, provided critical revision of the article for intellectual content, and approved final content.
